# Distribution of ExPEC Virulence Factors, *bla*_CTX-M_, *fos*A3, and *mcr*-1 in *Escherichia coli* Isolated From Commercialized Chicken Carcasses

**DOI:** 10.3389/fmicb.2018.03254

**Published:** 2019-01-14

**Authors:** Paula Signolfi Cyoia, Vanessa Lumi Koga, Erick Kenji Nishio, Sébastien Houle, Charles M. Dozois, Kelly Cristina Tagliari de Brito, Benito Guimarães de Brito, Gerson Nakazato, Renata Katsuko Takayama Kobayashi

**Affiliations:** ^1^Department of Microbiology, Center of Sciences Biological, Universidade Estadual de Londrina, Londrina, Brazil; ^2^Institut Armand-Frappier, Institut National de la Recherche Scientifique, Laval, QC, Canada; ^3^Avian Health Laboratory & Technical Innovation, Institute of Veterinary Research Desiderio Finamor (IPVDF), Eldorado do Sul, Rio Grande do Sul, Brazil

**Keywords:** ESBL, multidrug-resistance, phylogenetic groups, CTX-M, fosfomycin

## Abstract

Pathogenic *Escherichia coli* found in humans and poultry carcasses harbor similar virulence and resistance genes. The present study aimed to analyze the distribution of extraintestinal pathogenic *E. coli* (ExPEC) virulence factors (VF), *bla*_CTX−M_ groups, *fos*A3, and *mcr*-1 genes in *E. coli* isolated from commercialized chicken carcasses in southern Brazil and to evaluate their pathogenic risk. A total of 409 *E. coli* strains were isolated and characterized for genes encoding virulence factors described in ExPEC. Results of antimicrobial susceptibility testing confirmed that the strains were resistant to β-lactams, fosfomycin, colistin, and others resistance groups. The highest prevalence of VFs was observed in isolates belonging to the CTX-M groups, especially the CTX-M-2 group, when compared to those in other susceptible strains or strains with different mechanisms of resistance. Furthermore, ESBL strains were found to be 1.40 times more likely to contain three to five ExPEC virulence genes than non-ESBL strains. Our findings revealed the successful conjugation between ESBL-producing *E. coli* isolated from chicken carcass and the *E. coli* recipient strain J53, which suggested that genetic determinants encoding CTX-M enzymes may have originated from animals and could be transmitted to humans via food chain. In summary, chicken meat is a potential reservoir of MDR *E. coli* strains harboring resistance and virulence genes that could pose serious risks to human public health.

## Introduction

Humans and warm-blooded animals naturally harbor bacteria in their intestines, such as *Escherichia coli*, which is usually a non-pathogenic commensal bacterium. However, *E. coli* could cause extraintestinal diseases, including urinary tract infection, septicemia and meningitis in humans or even colibacillosis in poultry, which is attributed to the acquisition of virulence factors (VFs) (Müller et al., 2016).

Extraintestinal pathogenic *E. coli* (ExPEC) strains are characterized by several VF, including adhesins, invasins, protectins, and toxins, as well as several uptake systems for essential nutrients, such as iron (iron-uptake systems) (Johnson et al., 2008b). Commensal and pathogenic *E. coli* can be classified under different phylogenetic groups, since the VF found in each of the varieties are distributed differently (Clermont et al., 2000). Most commensal strains belong to phylogenetic group A or B1, and ExPEC strains, which harbor more VFs than commensal strains, are assigned to phylogenetic group B2 or D (Tenaillon et al., 2010; Cyoia et al., 2015).

In addition to VFs, the spread of resistance elements among human pathogens may be related to the Enterobacteriaceae family, in which *E. coli* belongs. Among the Gram-negative bacteria that are resistant to antibiotics, those that produce CTX-M-type ESBLs represent a serious public health concern worldwide (Xie et al., 2016). In particular, most commonly detected CTX-M groups include CTX-M-1, CTX-M-2, CTX-M-8, CTX-M-9, and CTXM-25 (Saravanan et al., 2018).

The detection of plasmidial genes that are mainly related to antimicrobial resistance to fosfomycin and colistin represents another major health concern (Sato et al., 2013; McGann et al., 2016). Fosfomycin is used to treat urinary tract infections (UTI) that are mostly caused by Gram-negative and Gram-positive bacteria, which are highly prevalent in North America (Giancola et al., 2017), and have recently received research attention because of the rapid spread of multidrug-resistance. This resistance is related to a novel gene called *fos*A3, which has been reported in *E. coli* and *Klebsiella pneumonia* and is often detected in *bla*_CTX−M_ -producing and multidrug-resistant *E. coli* both in animals and in clinical isolates (Ho et al., 2013). Colistin is prescribed for the treatment of UTI and has been associated with many cases of resistance worldwide. Furthermore, renewed attention has been paid to the *mcr*-1 gene because it has been detected not only in clinical isolates but also in animal, food, and environmental samples (Fernandes et al., 2016; McGann et al., 2016; Rapoport et al., 2016; Skov and Monnet, 2016; Zeng et al., 2016).

Pathogenic *E. coli* found in humans and poultry carcass were found to harbor similar virulence and resistance genes in the plasmids (Stromberg et al., 2017). These findings raise the possibility that *E. coli* present in the intestinal tract of healthy individuals could acquire those genes from *E. coli* derived from chicken meat, which could act as a reservoir for bacteria harboring resistance genes (Manges and Johnson, 2012). Therefore, present study aimed to analyze the distribution of ExPEC VFs, *bla*_CTX−M_ groups, and the *fos*A3 and *mcr*-1 genes in *E. coli* isolated from chicken carcasses commercialized in southern Brazil (States of Paraná-PR, Santa Catarina-SC, and Rio Grande do Sul-RS).

## Materials and Methods

### Bacterial Isolates

*Escherichia coli* strains were isolated in the Basic and Applied Bacteriology Laboratory at Londrina State University (Biosafety level 2) from 98 commercial refrigerated chicken carcass (35 chicken carcasses from PR, 23 chicken carcasses from SC, and 40 chicken carcasses from RS), sold in southern Brazil from 2013 to 2014. Each chicken carcass was rinsed into the sterile packaging with 100 mL of Brain Heart Infusion (Himedia Laboratories Pvt. Ltd., Mumbai, India). After homogenization, 0.1 mL of the mixture was smeared onto MacConkey agar (Neogen Corporation Lansing, Michigan) and Violet Red Bile Lactose agar (Oxoid Ltd., Basingstoke, Hants, UK) by the pour plate method. Colonies suspected to be *E. coli* were confirmed by biochemical testing using EPM-MILi and Simmons Citrate agar (PROBAC, Brazil). After biochemical confirmation, one to five strains were collected from each chicken carcass and subsequently analyzed for the genotypic characteristics of ExPEC virulence factors and phenotypic resistance. Only strains that showed difference in those characteristics were selected for further analysis.

### Antimicrobial Susceptibility Test

Antimicrobial susceptibility testing was performed using the standard disk diffusion method recommended by the Clinical and Laboratory Standards Institute (CLSI, 2015). The following antimicrobial agents were used in the study: 5 μ g of ciprofloxacin; 10 μg of each of ampicillin, gentamicin, norfloxacin, and enrofloxacin; 30 μg of each of cefazolin, cefotaxime, cefoxitin, ceftazidime, tetracycline, nalidixic acid, and chloramphenicol; 300 μg of nitrofurantoin; 1.25/23.75μg of trimethoprim-sulfamethoxazole; 200 μg of fosfomycin; and 20/10 μg of amoxicillin-clavulanic acid (Oxoid Ltd., Basingstoke, Hants, UK). Strains resistant to third-generation cephalosporins were confirmed for ESBL production by double-disk diffusion testing between amoxicillin/clavulanate and cefotaxime or ceftazidime (Jacoby and Han, 1996) or by conducting a combination disc test using cefotaxime, cefotaxime + clavulanic acid (Becton Dickinson, Sparks, MD), ceftazidime, and ceftazidime + clavulanic acid (Becton Dickinson, Sparks, MD), following the CLSI recommendations. The positive strains in the phenotypic tests to ESBL production were screened for ESBL genes, and the strains resistant to fosfomycin were screened for the *fos*A3 gene. The *E. coli* isolate ATCC 25922 was used as a quality control during antimicrobial susceptibility testing. Results were interpreted based on the CLSI criteria.

### Detection of Antimicrobial Resistance Genes

ESBL-producing *E. coli* was characterized for ESBL genes encoding CTX-M (groups 1, 2, 8, 9, and 25), TEM, and SHV by Polymerase Chain Reaction (PCR) (Arlet and Philippon, 1991; Bedenić et al., 2001; Woodford et al., 2006). The presence of acquired fosfomycin resistance genes such as *fos*A3 was determined by PCR using specific primer sets (Sato et al., 2013). The strains were additionally tested for the presence of colistin resistance gene *mcr*-1 by PCR (Liu et al., 2016). PCR amplicons were visualized on 2.0% agarose gels stained with GelRed (Biotium, Hayward, CA, USA). After gel electrophoresis, the images were captured using Image Capture Systems (LPixImageHE).

### Conjugation Experiments

To verify whether the plasmid harboring *bla*_CTX−M_ resistance genes could be transferred between *E. coli* strains, the horizontal-transfer efficiencies of the *bla*_CTX−M_ genes were assessed by performing conjugation experiments between three selected strains harboring *bla*_CTX−M_ resistance genes. Volumes of cultures of each donor (ESBL-producing *E. coli* isolated from chicken carcass) and azide-resistant *E. coli J53*, recipient strain grown in Luria-Bertani broth (Difco Laboratories, Detroit, Mich) were mixed and incubated for 18–24 h at 37°C. Transconjugants were then selected on MacConkey agar containing 2 μg/mL cefotaxime (Sigma Chemical Co., St. Louis, MO) and 100 μg/mL sodium azide (Sigma Chemical Co., St. Louis, MO) and subsequently used for phylogenetic analysis and testing for the presence of *bla*_CTX−M_ genes (Xie et al., 2016).

### Phylogenetic Classification

*E. coli* strains were assigned to phylogenetic groups (A, B1, B2, or D) by PCR (Clermont et al., 2000). Each PCR reaction contained 1.25 U of Taq DNA polymerase (Life technologies, Rockville, MD) in 1 × PCR buffer (Life Technologies, Rockville, MD), 0.2 mM each dNTP, 2.5 mM MgCl_2_, and 1 μM each primer. PCR amplicons were visualized on 2.0% agarose gels stained with GelRed (Biotium, Hayward, CA, USA). After gel electrophoresis, the images were captured using Image Capture Systems (LPixImageHE).

### Virulence Genes

We surveyed five VF genes that are normally studied in ExPEC strains. The selected genes included: *iut*A (aerobactin siderophore receptor gene), *hly*F (putative avian hemolysin), *iss* (episomal increased serum survival gene), *iro*N (salmochelin siderophore receptor gene), and *omp*T (episomal outer membrane protease gene) (Johnson et al., 2008a). Each PCR reaction contained 1.25 U of Taq DNA polymerase (Life Technologies, Rockville, MD) in 1 × PCR buffer (Life Technologies, Rockville, MD), 0.2 mM each dNTP, 2.5 mM MgCl_2_, and 1 μM each primer. PCR amplicons were visualized on 2.0% agarose gels stained with GelRed (Biotium, Hayward, CA, USA). After gel electrophoresis, the images were captured using Image Capture Systems (LPixImageHE).

### Statistical Analysis

Frequencies of ExPEC virulence genes in ESBL-producing and non-ESBL-producing strains were compared by Fisher's exact test and Pearson's Chi-square test. The risk of ESBL-producing *E. coli* harboring more ExPEC genes than non-ESBL-producing *E. coli* at 95% confidence interval (95% CI) was determined by calculating the relative risk (RR). Statistically significant differences were considered at *p* < 0.05. The test was performed using the statistical software R version 3.5.1.

## Results

### Antimicrobial Resistance of *E. coli* From Poultry Carcasses

**Figure 1:**
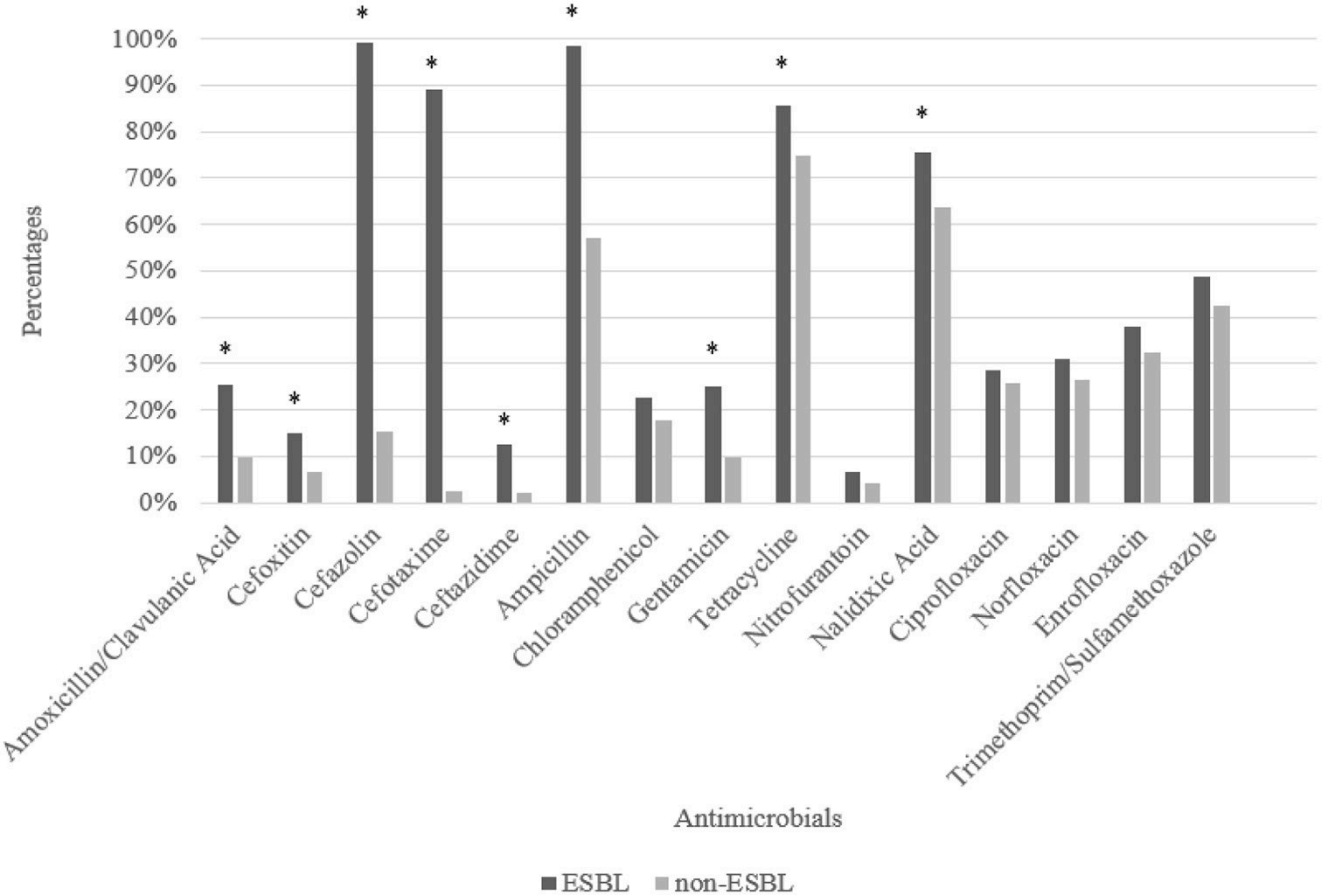
Percentage resistance exhibited by ESBL-producing *E. coli* strains and non-ESBL-producing *E. coli* strains isolated from commercial chicken carcasses in southern Brazil from 2013 to 2014. ^*^p < 0.05 by Pearson's Chi-square test.

A total of 409 *E. coli* isolates from chicken carcasses from southern Brazil were tested. Among these, 121, 135, and 153 were isolated from carcasses from the PR, SC, and RS states. Results of the antimicrobial susceptibility test indicated that strains from chicken carcasses showed a high frequency of antimicrobial resistance, in total 66% of the isolates were resistant to antibiotics. We identified multidrug-resistant *E. coli* strains from chicken carcasses from PR, SC and RS (82, 53, and 80%, respectively). The most common antimicrobial agents for which strains were found to be resistant included tetracycline (68.77%), nalidixic acid (67.61%), and ampicillin (68.77%). The ESBL phenotype was confirmed for 119 isolates (~32% of PR, 31% of SC, and 35% of RS) of the 409 strains isolated from commercial refrigerated chicken carcasses, which represents 29.1% of all isolates. Furthermore, ESBL-producing *E. coli* were found to be more resistant to a higher number of antimicrobials (*p* < 0.05) compared to non-ESBL-producing *E. coli* (Figure 1). Of the 409 *E. coli* strains tested, 99.3% were classified as susceptible to fosfomycin, whereas none showed intermediate resistance and three strains (0.70%) showed resistance to fosfomycin.

### Detection of Antimicrobial Resistance Genes

**Figure 2:**
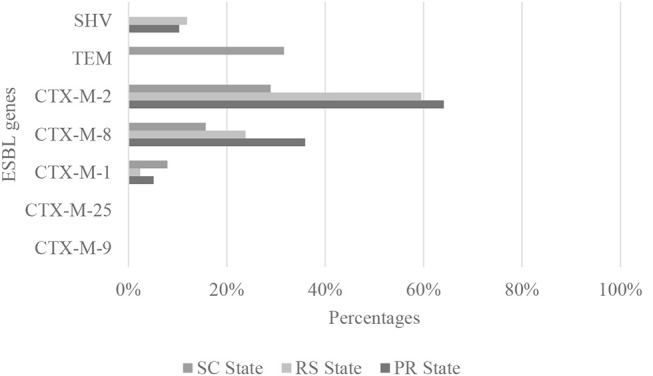
Distribution of ESBL genes encoding CTX-Ms, TEM, and SHV detected in *E. coli* strains isolated from commercial chicken carcasses in southern Brazil from 2013 to 2014.

The majority of ESBL-producing *E. coli* isolates (32.23%) were collected from the PR state, while the RS state showed the lowest number of ESBL-producing *E. coli* isolates (27.45%). Out of the 119 ESBL strains, 97 harbored the *bla*_CTX−M_ gene, six harbored CTX-M-1 group, 61 harbored CTX-M-2 group, and 30 harbored CTX-M-8 group (Table 1 and Figure 2). The CTX-M-9 group and CTX-M-25 group were not detected in the strains (Figure 2). The remaining *E. coli* strains harbored the *bla*_SHV_ (7.56%) and *bla*_TEM_ (10.08%) genes (Figure 2).

**Table 1:** Distribution of resistance and virulence genes among 119 ESBL-producing *E. coli* strains isolated from chicken carcasses commercialized in Brazil.

**β-lactamases, *fos*A3, *mcr-*1 genes**	**Virulence genes**	**Number of isolates**
Group 1 CTX-M	*hly*F, *omp*T, *iss, iro*N, *iut*A	1 PR
Group 1 CTX-M	*iut*A	1 RS
Group 1 CTX-M, Group 2 CTX-M	*hly*F, *omp*T, *iss, iro*N, *iut*A	1 PR
Group 1 CTX-M, Group 2 CTX-M	*hly*F, *omp*T, *iss, iut*A	1 SC
Group 1 CTX-M, Group 8 CTX-M, TEM	*iut*A	1 SC
Group 1 CTX-M, TEM	*hly*F, *omp*T, *iss, iro*N, *iut*A	1 SC
Group 2 CTX-M	*hly*F, *omp*T, *iss, iro*N, *iut*A	7 PR 2 SC 3 RS
Group 2 CTX-M	*omp*T, *iss, iro*N, *iut*A	1 RS
Group 2 CTX-M	*hly*F, *omp*T, *iro*N, *iut*A	1 RS
Group 2 CTX-M	*hly*F, *omp*T, *iss, iro*N	2 RS
Group 2 CTX-M	*hly*F, *omp*T, *iut*A	6 PR 2 SC 6 RS
Group 2 CTX-M	*iss, iut*A	1 RS
Group 2 CTX-M	*hly*F, *omp*T	1 RS
Group 2 CTX-M	*iro*N	1 RS
Group 2 CTX-M	*iut*A	8 PR 1 SC 3 RS
Group 2 CTX-M	None	2 SC 6 RS
Group 2 CTX-M, Group 8 CTX-M	None	1 PR
Group 2 CTX-M, Group 8 CTX-M, SHV, *fos*A3	*hly*F, *omp*T, *iss, iro*N, *iut*A	1 PR
Group 2 CTX-M, *mcr*-1	*hly*F, *omp*T, *iss, iro*N, *iut*A	1 PR
Group 2 CTX-M, TEM	*hly*F, *omp*T, *iro*N, *iut*A	1 SC
Group 2 CTX-M, TEM	*hly*F, *omp*T*, iut*A	1 SC
Group 2 CTX-M, TEM	*iut*A	1 SC
Group 8 CTX-M	*hly*F, *omp*T, *iss, iro*N, *iut*A	3 PR 1 SC 3 RS
Group 8 CTX-M	*hly*F, *omp*T, *iss, iro*N	2 SC 3 RS
Group 8 CTX-M	*hly*F, *omp*T, *iro*N, *iut*A	1 RS
Group 8 CTX-M	*hly*F, *omp*T	3 PR
Group 8 CTX-M	*hly*F	1 RS
Group 8 CTX-M	*omp*T	4 PR
Group 8 CTX-M	None	2 RS
Group 8 CTX-M, SHV	*hly*F, *omp*T, *iss, iro*N, *iut*A	2 PR
Group 8 CTX-M, TEM	*hly*F, *omp*T, *iss, iro*N, *iut*A	1 SC
Group 8 CTX-M, TEM	*iut*A	1 SC
SHV	*hly*F, *omp*T, *iss, iro*N, *iut*A	3 RS
SHV	*omp*T, *iss, iro*N, *iut*A	1 RS
SHV, *fos*A3	*hly*F, *omp*T, *iss, iro*N, *iut*A	1 PR
SHV, *mcr*-1	*hly*F, *omp*T, *iss, iro*N, *iut*A	1 RS
TEM	*hly*F, *omp*T, *iss, iut*A	1 SC
TEM	*hly*F, *iut*A	1 SC
TEM	*iro*N, *iut*A	1 SC
TEM	*iut*A	1 SC
TEM	None	1 SC
ND^a^, *fos*A3	None	1 SC
ND^a^, *mcr*-1	None	1 SC
ND^a^	*hly*F, *omp*T, *iss, iro*N, *iut*A	3 SC
ND^a^	*omp*T, *iss, iro*N, *iut*A	1 SC
ND^a^	*hly*F, *omp*T, *iss, iro*N	1 SC
ND^a^	*iss, iro*N, *iut*A	1 SC
ND^a^	*hly*F, *omp*T, *iut*A	1 RS
ND^a^	*iro*N, *iut*A	1 SC
ND^a^	*iut*A	5 SC
ND^a^	None	2 SC

*ND^a^, not detected*.

Fosfomycin resistance was identified based on phenotypic tests and subsequently confirmed by PCR. The three fosfomycin-resistant strains that harbored the *fos*A3 gene were found to be *bla*_CTX−M_ positive (3.33%). PCR analysis of the 119 ESBL-producing *E. coli* isolates revealed that 2.50% of the isolates harbored genes encoding resistance to colistin, corresponding to one resistant strain from each state (PR, SC, and RS). Furthermore, these strains were ESBL-producing *E. coli*, and two of these strains harbored five ExPEC virulence genes tested in the present study (*iss, iro*N, *iut*A, *hly*F, and *omp*T) (Table 1) and were assigned to different phylogenetic groups (A, B2, and B1).

### Conjugation Experiments

Among the *bla*_CTX−M_ positive *E. coli* isolates tested that belonged to phylogenetic group B1, all strains successfully transferred their cefotaxime resistance phenotypes to the *E. coli* recipient strain J53 via conjugation.

### Phylogenetic Classification

Phylogenetic analysis revealed that most of the *E. coli* strains belonged to group B1 (36.6%), followed by groups A (31.7%), D (28.1%), and B2 (3.40%) (Table 2). The determination of *E. coli* phylogenetic groups showed that the majority of the 119 ESBL-producing *E. coli* belonged to phylogenetic group D (36.06%), followed by a nearly even distribution of the remaining three phylogenetic groups, namely, B1 (31.97%), A (27.63%), and B2 (4.22%) (Table 2).

**Table 2:** Phylogenetic distribution of 290 non-ESBL-producing *E. coli* strains and 119 ESBL-producing *E. coli* strains isolated from chicken carcasses from different southern Brazilian states.

**Phylogenetic groups**	**Southern Brazilian states—N°of strains (%)**								
	**PR**			**SC**			**RS**		
**(N°of strains)**	**Non-ESBL**	**ESBL**	**Total**	**Non-ESBL**	**ESBL**	**Total**	**Non-ESBL**	**ESBL**	**Total**
A (130)	24 (29.3)	11 (28.2)	35 (28.9)	39 (40.2)	11 (28.9)	50 (37.0)	34 (30.6)	11 (26.2)	45 (29.4)
B1 (150)	33 (40.2)	12 (30.8)	45 (37.2)	33 (34.0)	13 (34.2)	46 (34.1)	46 (41.4)	13 (30.9)	59 (38.6)
B2 (14)	4 (4.9)	1 (2.6)	5 (4.1)	1 (1.0)	2 (5.3)	3 (2.2)	4 (3.6)	2 (4.8)	6 (3.9)
D (115)	21 (25.6)	15 (38.5)	36 (29.8)	24 (24.7)	12 (31.6)	36 (26.7)	27 (24.3)	16 (38.1)	43 (28.1)
	82 (100)	39 (100)	121 (100)	97 (100)	38 (100)	135 (100)	111 (100)	42 (100)	153 (100)

### Virulence Genes

ExPEC VFs were identified in the various *E. coli* strains. Among the 409 *E. coli* strains analyzed, the prevalence of individual ExPEC VF genes ranged from 33.3% (*iss*, an episomal increased serum survival gene) to 51.6% (*iut*A, an aerobactin siderophore receptor gene). Results indicated that 58% of ESBL-producing *E. coli* harbored three to five ExPEC virulence genes (Table 1).

The highest prevalence of ExPEC VFs was observed in strains harboring CTX-M resistance relative to other susceptible strains or even strains with different mechanisms of resistance (*p* < 0.01). The relative risk for ESBL strains that did not contain any ExPEC genes was 0.35 (95 % CI, 0.21–0.57; *p* < 0.01). On the other hand, the RR for ESBL strains harboring three or more ExPEC genes was 1.40 (95 % CI, 1.13–1.73; *p* < 0.01) (Table 3). For each non-ESBL strain harboring three or more ExPEC virulence genes (Supplementary Material), there are 1.40 ESBL strains harboring three or more ExPEC virulence genes (RR>1). For example, in the PR state, the *iut*A gene was present in 54% of the *E. coli* isolates, and present in 80% of the *bla*_CTX−M_ producing *E. coli*. Similar results were observed in the other two states for all five virulence genes.

**Table 3:** Risk factor analysis indicating that ESBL-producing *E. coli* harbor more virulence genes than non-ESBL-producing *E. coli*.

**ExPEC virulence genes**	**ESBL *n* = 119**	**Non-ESBL *n* = 290**	**Relative risk (95% CI)**	**^a^*p*-value**
			
None	15	105	0.35 (0.21–0.57)	<0.01
1 gene	28	52	1.31 (0.87–1.97)	0.195
2 genes	8	15	1.29 (0.57–2.98)	0.537
3–5 genes	68	118	1.40 (1.13–1.73)	<0.01

a *p < 0.05 by Fisher's exact test and Pearson's Chi-square test*.

## Discussion

In the present study, we analyzed a total of 409 *E. coli* strains from commercial chicken carcasses in Brazil isolated from 2013 to 2014. About 71% of isolates were MDR (Magiorakos et al., 2012), which demonstrate the high antimicrobial resistance. Our current findings are consistent with reports from other countries, which detected MDR in Gram negative bacteria from chicken meat in Italy (66.9% resistant) and India (79.6% resistant) (Ghodousi et al., 2015; Shrestha et al., 2017). In the states of PR and RS, approximately 80% of carcasses were found to be contaminated with *E. coli* that were resistant to three or more antimicrobial groups, whereas the rates of resistance in the state of SC were slightly lower (53%). The higher rates of antimicrobial resistance and MDR in strains could be due to environmental contamination with antibiotic residues in aviculture industries and/or selective pressure caused by the indiscriminate use of antimicrobial compounds as a result of poor monitoring by regulatory bodies (Koga et al., 2015). Importantly, some growth promoters, such as poultry feeds, have been prohibited in animal production in several countries, like in Brazil since 1998 (Brasil Ministério da Agricultura, 2003, 2009).

Almost 30% of the isolates analyzed in the present study were found to be resistant to β-lactams and thus represent a potential health concern. The resistant *E. coli* harbored genes encoding ESBL enzymes that hydrolyze penicillins, cephalosporins, and monobactams and were inhibited by treatment with “classical” β-lactamase inhibitors such as clavulanic acid, sulbactam, and tazobactam (Bevan et al., 2017; Saravanan et al., 2018). Notably, ESBL-producing *E. coli* showed stronger resistance to others antimicrobials, such as aminoglycosides, quinolones, and tetracyclines, when compared to non-ESBL-producing *E. coli* (*p* < 0.05), further promoting the health risks due to consumption of undercooked meat or the handling or preparation of uncooked poultry products contaminated with resistant strains (Shrestha et al., 2017; Saravanan et al., 2018). CTX-M ß-lactamases are the most widespread type of ESBL and have been identified since the mid-2000s and were specifically detected in clinical isolates of *E. coli* (Bush, 2018). ESBL-producing bacteria have been increasingly detected in meat from food-producing animals such as, poultry (Ghodousi et al., 2015; Shrestha et al., 2017; Poirel et al., 2018). Our findings have raised significant concerns, since the 30% prevalence of ESBL-producing samples in chicken carcasses in southern Brazil was higher than those reported in other regions, as in USA (27%), in India (21%) and in other samples from Brazil (7%) (Freeman et al., 2009; Datta et al., 2014; Gonçalves et al., 2016). Among all ESBL strains, we found 97% classified as *bla*_CTX−M_ and the majority belonged to CTX-M-2 group, although the rates varied depending on the region worldwide. Recent studies reported the presence of the CTX-M-1 resistance genes in *E. coli* strains from poultry meat from Sweden (54–58%), Belgium (62%), Canada (66.2%), Italy (8.9%), and Japan (34%) (Smet et al., 2010; Denisuik et al., 2013; Brolund et al., 2014; Ghodousi et al., 2015; Nahar et al., 2018). However, CTX-M-9 represented the most prevalent group in reports of ESBL *E. coli* from Spain (Garrido et al., 2014), Portugal (Fernandes et al., 2014), Japan (Nahar et al., 2018), and Italy (Ghodousi et al., 2015).

One important finding from the current study is the successful conjugation between ESBL-producing *E. coli* isolated from chicken carcass to the *E. coli* recipient strain J53, which suggest that genetic determinants encoding CTX-M enzymes could be conjugative. According to Xie et al. (2016), commensal B1 strains isolated from food-producing animals could act as reservoirs of ESBL genes, which could be disseminated to human bacteria via the food chain, thereby raising a significant public health concern (Leverstein-van Hall et al., 2011; Xie et al., 2016; Poirel et al., 2018). Furthermore, resistance conferred by ESBLs is often associated with resistance to other classes of antibiotics, such as trimethoprim-sulfamethoxazole, aminoglycosides, and fluoroquinolone (Coque et al., 2008; Zeng and Lin, 2017). Therefore, the transfer of CTX-M mobile plasmids are likely to be accompanied by acquisition of other resistance genes. Some studies reported that plasmid-mediated fosfomycin resistance is frequently detected among CTX-M-producing *E. coli* isolated from food-producing animals (Sato et al., 2013; Xie et al., 2016). During sample collection in 2013, fosfomycin was not commonly used in animal production because of its high cost; nevertheless, 3% of the strains tested positive for the presence of the *fos*A3 gene.

The use of polymyxins (colistin) in food-producing animals, especially in feed additives, represents another health concern. One colistin-resistant *E. coli* strain harboring five ExPEC virulence genes was detected in each of the southern Brazilian states. Several recent studies have also suggested the possibility of transfer of the *mcr*-1 gene to humans via the food chain (Carnevali et al., 2016; Wang et al., 2017). Although the current results indicated a very low presence of the *mcr*-1 gene, other studies indicated that the higher prevalence of colistin resistance could be attributed to the widespread use of colistin in food production in recent years (Huang et al., 2017). Thus, the use of fosfomycin and colistin in food production, such as in poultry, could lead to a public health concern, considering that these antimicrobials are used for the treatment of extraintestinal infections in humans. Therefore, similar to colistin, fosfomycin should also be banned from animal production in many countries.

Current evidence indicates that *E. coli* isolated from chickens and human ExPECs, harbor highly similar virulence genes, thereby suggesting a potential risk to cause diseases in humans (Manges and Johnson, 2012). A higher number of virulence factors present in ExPEC indicates a link to pathogenicity (Pitout, 2012). Furthermore, studies demonstrated an association between ExPEC virulence factors and phylogenetic groups. Intestinal *E. coli* isolates belonging to groups A and B1 harbor fewer ExPEC virulence genes, and ExPECs strains belonging to groups B2 and D contain a higher number of virulence genes (Koga et al., 2015; Müller et al., 2016; Pavlickova et al., 2017). Consistent with previous studies, most *E. coli* strains isolated from chicken carcasses harbor three to five ExPEC virulence genes (33–51%, varying between the five genes) and belonged to phylogenetic group B1 (36%), which represents a group of more multi-resistant commensal strains (Koga et al., 2015; Müller et al., 2016). Among these strains, 58% of ESBL-producing *E. coli* harbored three to five ExPEC virulence genes. Most of these strains were associated with phylogenetic group D, unlike non-ESBL-producing *E. coli*, which were associated with group B1. These rates are high compared to 28% of ExPEC isolated from patients mostly with UTIs in southern Brazil (Cyoia et al., 2015) or very similar to those reported in APEC strains (Mohamed et al., 2018), thereby indicating that some ESBL-producing *E. coli* strains from poultry meat are potentially pathogenic.

Importantly, *bla*_CTX−M_ ESBL-producing *E. coli* strains were found to harbor a higher number of ExPEC virulence genes relative to other susceptible strains or even strains that were resistant to other groups of antimicrobials (*p* < 0.01). In addition, ESBL strains are 1.40 times more likely to contain three to five ExPEC virulence genes than non-ESBL strains, which in turn increases their risk for pathogenic potential (RR = 1.40, 95% CI, 1.13–1.73; *p* < 0.01). The above findings suggest that *E. coli* present in chicken meat, which could act as a reservoir for these antimicrobial resistance and virulence genes could be a potential risk for colonization and/or transfer of this resistance to bacteria in the intestinal tracts of humans.

Despite the importance of identifying ESBL-producing *E. coli* belonging to phylogenetic group D, which is commonly associated with strains found in hospitals and ambulatory patients (Pietsch et al., 2017), the detection of commensal strains from group B1 is also notable. Although transferable isolates belonging to phylogenetic group B1 do not comprise the most virulent phylogenetic group (such as B2 or D), these strains still harbor both virulence and resistance genes. Therefore, chicken meat could serve as an important reservoir for resistance genes and could be responsible for the spread of MDR bacteria via the food chain.

## Conclusion

Our results highlight the high prevalence of ExPEC virulence genes and antimicrobial resistance genes associated with chicken meat. Brazil is the largest exporter of chicken meat and the second largest producer of chicken meat worldwide. These findings further represent a public health concern, considering that chicken meat could serve as a reservoir for the spread of plasmids harboring resistance and virulence genes through the food chain. Future studies should investigate whether both, resistance and virulence genes are transferred together to other bacteria and determine whether they are present in the same plasmid.

## Author Contributions

PC contributed to the development of experimental research, data analysis, and preparation of the article. VK, BB, and KB contributed to the development of experimental research. EN contributed to the statistical analysis. RK, GN, KB, BB, SH, and CD contributed to and assisted in the design of the work, assisted in critical data interpretation, and in preparation of the article. All authors have participated in this study and commented on the manuscript.

### Conflict of Interest Statement

The authors declare that the research was conducted in the absence of any commercial or financial relationships that could be construed as a potential conflict of interest.
