# Supplementary material for: Distribution of ExPEC Virulence Factors, blaCTX-M, fosA3, and mcr-1 in Escherichia coli Isolated From Commercialized Chicken Carcasses
Source: Front Microbiol. 2019 Jan 14;9:3254. doi: 10.3389/fmicb.2018.03254 (PMC6339928; doi:10.3389/fmicb.2018.03254)
Supplement: Supplementary file 1 [file Table_1.DOCX]

Supplementary data Table| **Distribution of virulence genes among non-ESBL-producing *E. coli* strains isolated from chicken carcasses commercialized in Brazil.**

| **Isolate number** | **Virulence genes** | | | | |
| --- | --- | --- | --- | --- | --- |
| PR 1.1 | *iut*A |  |  |  |  |
| PR 1.2 | None |  |  |  |  |
| PR 1.3 | None |  |  |  |  |
| PR 2.9 A | None |  |  |  |  |
| PR 8.1 | None |  |  |  |  |
| PR 8.2 A | None |  |  |  |  |
| PR 9.1 | *omp*T | *iss* | *iro*N | *iut*A |  |
| PR 9.2 | *iut*A |  |  |  |  |
| PR 9.3 | *iut*A |  |  |  |  |
| PR 9.4 | None |  |  |  |  |
| PR 9.5 | *hly*F | *omp*T |  |  |  |
| PR 10.2 | *hly*F | *omp*T |  |  |  |
| PR 10.3 | *hly*F | *omp*T |  |  |  |
| PR 10.4 | *omp*T |  |  |  |  |
| PR 10.5 | None |  |  |  |  |
| PR 20.8 | *omp*T |  |  |  |  |
| PR 20 EC | *iss* |  |  |  |  |
| PR 20 A | *iss* |  |  |  |  |
| PR 20 C | None |  |  |  |  |
| PR 21.1 | None |  |  |  |  |
| PR 21.9 | None |  |  |  |  |
| PR 21 A | None |  |  |  |  |
| PR 21 C | None |  |  |  |  |
| PR 23.2 | *hly*F | *omp*T | *iss* | *iut*A |  |
| PR 23.3 | None |  |  |  |  |
| PR 23.4 | *hly*F | *omp*T | *iss* | *iut*A |  |
| PR 23 EC | *hly*F | *omp*T | *iss* | *iro*N |  |
| PR 24.2 | *hly*F | *omp*T | *iss* | *iro*N |  |
| PR 24.5 | None |  |  |  |  |
| PR 24.6 | *hly*F | *omp*T | *iss* |  |  |
| PR 25.1 | *hly*F | *omp*T | *iut*A |  |  |
| PR 25 EC | *hly*F | *omp*T | *iut*A |  |  |
| PR 26.1 | None |  |  |  |  |
| PR 26.2 | *hly*F | *omp*T | *iss* | *iro*N |  |
| PR 26.3 | *hly*F | *omp*T | *iut*A |  |  |
| PR 26.5 | *hly*F | *omp*T | *iss* | *iro*N | *iut*A |
| PR 27.1 | *hly*F | *omp*T | *iss* | *iro*N | *iut*A |
| PR 27.2 | *hly*F | *omp*T | *iss* | *iro*N | *iut*A |
| PR 28.2 | *hly*F | *omp*T | *iss* | *iro*N | *iut*A |
| PR 28.3 | None |  |  |  |  |
| PR 28EC | None |  |  |  |  |
| PR 28 A | *hly*F | *omp*T | *iss* | *iro*N | *iut*A |
| PR 28 C | *iut*A |  |  |  |  |
| PR 28 T | *iss* | *iut*A |  |  |  |
| PR 29.4 | *iut*A |  |  |  |  |
| PR 29EC | None |  |  |  |  |
| PR 29 A | None |  |  |  |  |
| PR 29TE | *hly*F | *omp*T | *iss* | *iut*A |  |
| PR 30.5 | *iut*A |  |  |  |  |
| PR 30 A | *iut*A |  |  |  |  |
| PR 30 C | *iut*A |  |  |  |  |
| PR 30TE | *iut*A |  |  |  |  |
| PR 32.1 | None |  |  |  |  |
| PR 32.1 A | None |  |  |  |  |
| PR 32.2 A | *hly*F | *omp*T | *iut*A |  |  |
| PR 33.1 | None |  |  |  |  |
| PR 33.2 | *iut*A |  |  |  |  |
| PR 33.3 | *hly*F | *omp*T | *iut*A |  |  |
| PR 33.5 | *iut*A |  |  |  |  |
| PR 33 A | *hly*F | *omp*T | *iss* | *iro*N | *iut*A |
| PR 33 C | None |  |  |  |  |
| PR 33.1TE | *hly*F | *omp*T | *iss* | *iro*N |  |
| PR 34.2 | None |  |  |  |  |
| PR 34.1EC | None |  |  |  |  |
| PR 34.2EC | *hly*F | *omp*T | *iss* | *iro*N |  |
| PR 34.1 A | *iss* |  |  |  |  |
| PR 34.2 A | None |  |  |  |  |
| PR 34.1TE | None |  |  |  |  |
| PR 35.3 | None |  |  |  |  |
| PR 35.5 | *hly*F | *omp*T | *iss* | *iro*N | *iut*A |
| PR 35 EC | *hly*F | *omp*T | *iss* | *iro*N | *iut*A |
| PR 36.1 | *hly*F | *omp*T | *iss* | *iro*N |  |
| PR 36.4 | *hly*F | *omp*T | *iss* | *iro*N |  |
| PR 41.1 | None |  |  |  |  |
| PR 41.1TE | *hly*F | *omp*T | *iro*N | *iut*A |  |
| PR 41.2TE | *hly*F | *omp*T | *iro*N | *iut*A |  |
| PR 42.3 | None |  |  |  |  |
| PR 42.4 | *hly*F | *omp*T | *iss* | *iut*A |  |
| PR 42 C | *hly*F | *omp*T | *iss* | *iut*A |  |
| PR 43.5 | *iut*A |  |  |  |  |
| PR 44.5 | None |  |  |  |  |
| PR 44.2 A | *iut*A |  |  |  |  |
| SC 1 | *hly*F | *omp*T | *iss* | *iut*A | *iro*N |
| SC 2 | *hly*F | *omp*T | *iro*N |  |  |
| SC 3 | *hly*F | *omp*T | *iro*N |  |  |
| SC 4 | None |  |  |  |  |
| SC 5 | None |  |  |  |  |
| SC 6 | None |  |  |  |  |
| SC 7 | *hly*F | *omp*T | *iut*A | *iro*N |  |
| SC 8 | *hly*F | *omp*T | *iss* | *iut*A | *iro*N |
| SC 10 | None |  |  |  |  |
| SC 12 | *hly*F |  |  |  |  |
| SC 13 | *iut*A | *iro*N |  |  |  |
| SC 15 | *hly*F |  |  |  |  |
| SC 16 | *omp*T |  |  |  |  |
| SC 17 | *omp*T |  |  |  |  |
| SC 19 | *hly*F | *omp*T | *iss* | *iro*N |  |
| SC 21 | None |  |  |  |  |
| SC 24 | *iut*A | *iro*N |  |  |  |
| SC 27 | *iut*A |  |  |  |  |
| SC 31 | *hly*F | *omp*T | *iut*A |  |  |
| SC 35 | *iut*A |  |  |  |  |
| SC 36 | *iut*A |  |  |  |  |
| SC 37 | None |  |  |  |  |
| SC 47 | *hly*F | *omp*T | *iut*A | *iro*N |  |
| SC 48 | *hly*F | *omp*T | *iss* | *iut*A | *iro*N |
| SC 60 | *iut*A |  |  |  |  |
| SC 61 | *iut*A |  |  |  |  |
| SC 63 | *hly*F | *omp*T | *iss* | *iro*N |  |
| SC 64 | *iro*N |  |  |  |  |
| SC 66 | None |  |  |  |  |
| SC 67 | *omp*T | *iss* | *iut*A | *iro*N |  |
| SC 68 | *iut*A |  |  |  |  |
| SC 69 | *iut*A |  |  |  |  |
| SC 70 | None |  |  |  |  |
| SC 71 | *iut*A |  |  |  |  |
| SC 72 | *iut*A |  |  |  |  |
| SC 77 | *iut*A |  |  |  |  |
| SC 78 | None |  |  |  |  |
| SC 80 | *hly*F | *omp*T |  |  |  |
| SC 81 | *hly*F | *omp*T | *iut*A | *iro*N |  |
| SC 83 | *hly*F | *omp*T | *iut*A | *iro*N |  |
| SC 84 | *hly*F | *omp*T | *iut*A | *iro*N |  |
| SC 86 | *hly*F | *omp*T | *iut*A | *iro*N |  |
| SC 87 | *hly*F | *omp*T | *iut*A | *iro*N |  |
| SC 89 | None |  |  |  |  |
| SC 90 | *omp*T | *iss* |  |  |  |
| SC 92 | *hly*F | *omp*T | *iss* | *iut*A | *iro*N |
| SC 93 | *hly*F | *omp*T | *iss* | *iut*A | *iro*N |
| SC 95 | *hly*F | *omp*T | *iss* | *iut*A | *iro*N |
| SC 96 | *hly*F | *omp*T | *iss* | *iut*A | *iro*N |
| SC 98 | *iut*A |  |  |  |  |
| SC 99 | *hly*F | *omp*T | *iss* | *iro*N |  |
| SC 101 | *hly*F | *omp*T | *iss* | *iro*N |  |
| SC 103 | *hly*F | *iro*N |  |  |  |
| SC 104 | None |  |  |  |  |
| SC 105 | *iut*A |  |  |  |  |
| SC 107 | *hly*F | *omp*T | *iss* | *iro*N |  |
| SC 108 | *omp*T | *iss* | *iro*N |  |  |
| SC 110 | *hly*F | *omp*T | *iss* | *iut*A | *iro*N |
| SC 111 | *hly*F | *omp*T | *iss* | *iut*A | *iro*N |
| SC 112 | None |  |  |  |  |
| SC 114 | None |  |  |  |  |
| SC 115 | None |  |  |  |  |
| SC 117 | None |  |  |  |  |
| SC 119 | *hly*F | *omp*T | *iss* | *iut*A | *iro*N |
| SC 120 | None |  |  |  |  |
| SC 121 | None |  |  |  |  |
| SC 126 | None |  |  |  |  |
| SC 127 | *iro*N |  |  |  |  |
| SC 130 | None |  |  |  |  |
| SC 137 | *iut*A |  |  |  |  |
| SC 138 | None |  |  |  |  |
| SC 140 | None |  |  |  |  |
| SC 143 | None |  |  |  |  |
| SC 144 | None |  |  |  |  |
| SC 151 | None |  |  |  |  |
| SC 155 | *iut*A |  |  |  |  |
| SC 156 | *iut*A |  |  |  |  |
| SC 158 | *hly*F | *omp*T | *iut*A |  |  |
| SC 159 | *hly*F | *omp*T | *iut*A |  |  |
| SC 160 | *hly*F | *omp*T | *iut*A |  |  |
| SC 165 | *hly*F | *omp*T | *iut*A |  |  |
| SC 166 | None |  |  |  |  |
| SC 168 | *hly*F | *omp*T | *iss* | *iro*N |  |
| SC 172 | *hly*F | *omp*T |  |  |  |
| SC 173 | *hly*F | *omp*T |  |  |  |
| SC 178 | *hly*F | *omp*T | *iut*A |  |  |
| SC 179 | None |  |  |  |  |
| SC 181 | None |  |  |  |  |
| SC 182 | *hly*F | *omp*T | *iut*A |  |  |
| SC 184 | *hly*F | *omp*T | *iut*A | *iro*N |  |
| SC 185 | *hly*F | *omp*T | *iut*A | *iro*N |  |
| SC 187 | None |  |  |  |  |
| SC 188 | None |  |  |  |  |
| SC 189 | None |  |  |  |  |
| SC 193 | *hly*F | *omp*T | *iut*A | *iro*N |  |
| SC 194 | *hly*F | *omp*T | *iut*A | *iro*N |  |
| RS 1 | *hlyF* | *ompT* | *iutA* | *iroN* | *iss* |
| RS 11 | *ompT* | *iutA* | *iroN* | *iss* |  |
| RS 12 | *hlyF* | *ompT* | *iutA* |  |  |
| RS 19 | *iutA* | *iroN* |  |  |  |
| RS 29 | None |  |  |  |  |
| RS 30 | None |  |  |  |  |
| RS 31 | *ompT* |  |  |  |  |
| RS 32 | *hlyF* | *ompT* | *iroN* | *iss* |  |
| RS 37 | None |  |  |  |  |
| RS 43 | *iutA* |  |  |  |  |
| RS 46 | *hlyF* | *ompT* | *iutA* | *iroN* | *iss* |
| RS 48 | *hlyF* | *ompT* | *iutA* | *iroN* | *iss* |
| RS 49 | *iutA* |  |  |  |  |
| RS 60 | *hlyF* | *ompT* | *iroN* | *iss* |  |
| RS 63 | *hlyF* | *ompT* | *iutA* | *iroN* | *iss* |
| RS 65 | *hlyF* | *ompT* | *iutA* | *iroN* | *iss* |
| RS 71 | *hlyF* | *ompT* | *iroN* | *iss* |  |
| RS 73 | None |  |  |  |  |
| RS 75 | *hlyF* | *ompT* | *iutA* | *iroN* | *iss* |
| RS 81 | None |  |  |  |  |
| RS 82 | None |  |  |  |  |
| RS 85 | None |  |  |  |  |
| RS 94 | *iutA* |  |  |  |  |
| RS 104 | None |  |  |  |  |
| RS 112 | None |  |  |  |  |
| RS 115 | *hlyF* | *ompT* | *iroN* | *iss* |  |
| RS 118 | *hlyF* | *ompT* | *iutA* |  |  |
| RS 121 | None |  |  |  |  |
| RS 128 | None |  |  |  |  |
| RS 131 | None |  |  |  |  |
| RS 132 | *hlyF* | *ompT* | *iutA* |  |  |
| RS 134 | *iutA* |  |  |  |  |
| RS 135 | *iroN* |  |  |  |  |
| RS 136 | *hlyF* | *ompT* | *iroN* | *iss* |  |
| RS 145 | *hlyF* | *ompT* | *iroN* | *iss* |  |
| RS 148 | None |  |  |  |  |
| RS 156 | *hlyF* | *ompT* | *iutA* | *iroN* | *iss* |
| RS 158 | *hlyF* | *ompT* | *iss* |  |  |
| RS 165 | None |  |  |  |  |
| RS 166 | *hlyF* | *ompT* | *iutA* | *iroN* | *iss* |
| RS 171 | *hlyF* | *ompT* | *iroN* | *iss* |  |
| RS 172 | *hlyF* | *iutA* | *iroN* |  |  |
| RS 173 | *hlyF* | *ompT* | *iutA* | *iroN* | *iss* |
| RS 183 | *hlyF* | *ompT* | *iroN* | *iss* |  |
| RS 185 | *iutA* |  |  |  |  |
| RS 188 | None |  |  |  |  |
| RS 196 | None |  |  |  |  |
| RS 205 | *hlyF* | *ompT* | *iutA* | *iroN* |  |
| RS 206 | *hlyF* | *ompT* | *iroN* | *iss* |  |
| RS 207 | *iroN* |  |  |  |  |
| RS 208 | *hlyF* | *ompT* | *iroN* | *iss* |  |
| RS 209 | *hlyF* | *ompT* | *iutA* |  |  |
| RS 214 | None |  |  |  |  |
| RS 221 | None |  |  |  |  |
| RS 222 | None |  |  |  |  |
| RS 225 | *hlyF* | *ompT* | *iutA* | *iroN* |  |
| RS 231 | None |  |  |  |  |
| RS 232 | *iss* |  |  |  |  |
| RS 233 | None |  |  |  |  |
| RS 236 | *hlyF* | *ompT* | *iutA* |  |  |
| RS 239 | *hlyF* | *ompT* | *iroN* | *iss* |  |
| RS 279 | *iutA* | *iroN* |  |  |  |
| RS 280 | *hlyF* | *iss* |  |  |  |
| RS 281 | *hlyF* | *ompT* | *iutA* | *iroN* | *iss* |
| RS 283 | *hlyF* | *ompT* | *iroN* | *iss* |  |
| RS 284 | *ompT* | *iroN* | *iss* |  |  |
| RS 285 | None |  |  |  |  |
| RS 288 | None |  |  |  |  |
| RS 291 | None |  |  |  |  |
| RS 292 | None |  |  |  |  |
| RS 295 | None |  |  |  |  |
| RS 301 | None |  |  |  |  |
| RS 312 | *hlyF* | *ompT* | *iutA* | *iroN* | *iss* |
| RS 317 | None |  |  |  |  |
| RS 320 | *hlyF* | *ompT* | *iroN* | *iss* |  |
| RS 321 | None |  |  |  |  |
| RS 327 | *hlyF* | *ompT* | *iutA* | *iroN* | *iss* |
| RS 328 | *hlyF* | *ompT* | *iroN* | *iss* |  |
| RS 333 | *iutA* |  |  |  |  |
| RS 343 | None |  |  |  |  |
| RS 344 | *hlyF* | *ompT* | *iroN* | *iss* |  |
| RS 351 | None |  |  |  |  |
| RS 354 | *hlyF* | *ompT* | *iutA* | *iroN* |  |
| RS 357 | None |  |  |  |  |
| RS 362 | *iutA* |  |  |  |  |
| RS 434 | None |  |  |  |  |
| RS 437 | None |  |  |  |  |
| RS 440 | *hlyF* | *ompT* | *iutA* | *iroN* |  |
| RS 448 | None |  |  |  |  |
| RS 454 | None |  |  |  |  |
| RS 455 | None |  |  |  |  |
| RS 457 | None |  |  |  |  |
| RS 458 | *hlyF* | *ompT* | *iroN* | *iss* |  |
| RS 459 | *hlyF* | *ompT* | *iroN* | *iss* |  |
| RS 462 | *hlyF* | *ompT* | *iutA* | *iroN* | *iss* |
| RS 463 | *hlyF* | *ompT* | *iutA* |  |  |
| RS 465 | *hlyF* | *ompT* | *iutA* | *iroN* | *iss* |
| RS 468 | None |  |  |  |  |
| RS 470 | *hlyF* | *ompT* | *iutA* |  |  |
| RS 471 | *hlyF* | *ompT* | *iutA* | *iroN* | *iss* |
| RS 472 | None |  |  |  |  |
| RS 485 | None |  |  |  |  |
| RS 488 | *hlyF* | *ompT* | *iutA* |  |  |
| RS 491 | *hlyF* | *ompT* |  |  |  |
| RS 497 | None |  |  |  |  |
| RS 498 | *hlyF* | *ompT* | *iutA* | *iroN* | *iss* |
| RS 500 | *hlyF* | *ompT* | *iutA* | *iroN* |  |
| RS 501 | *hlyF* | *ompT* | *iroN* | *iss* |  |
| RS 507 | *hlyF* | *ompT* | *iutA* | *iroN* | *iss* |
| RS 508 | None |  |  |  |  |
| RS 509 | *hlyF* | *ompT* | *iutA* | *iroN* |  |
| RS 511 | *hlyF* | *ompT* | *iutA* | *iroN* | *iss* |
